# Modern Rehabilitation Technologies of Patients with Motor Disorders at an Early Rehabilitation of Stroke (Review)

**DOI:** 10.17691/stm2022.14.6.07

**Published:** 2022-11-28

**Authors:** A.E. Khrulev, K.M. Kuryatnikova, А.N. Belova, P.S. Popova, S.Е. Khrulev

**Affiliations:** Associate Professor, Department of Nervous Diseases, Deputy Director of the Rehabilitation Institute, University Clinic; Privolzhsky Research Medical University, 10/1 Minin and Pozharsky Square, Nizhny Novgorod, 603005, Russia; 5-Year Student, Faculty of General Medicine; Privolzhsky Research Medical University, 10/1 Minin and Pozharsky Square, Nizhny Novgorod, 603005, Russia; Professor, Head of the Department of Medical Rehabilitation; Privolzhsky Research Medical University, 10/1 Minin and Pozharsky Square, Nizhny Novgorod, 603005, Russia; 5-Year Student, Faculty of General Medicine; Privolzhsky Research Medical University, 10/1 Minin and Pozharsky Square, Nizhny Novgorod, 603005, Russia; Director of the Rehabilitation Institute, University Clinic; Privolzhsky Research Medical University, 10/1 Minin and Pozharsky Square, Nizhny Novgorod, 603005, Russia

**Keywords:** cerebral stroke, motor disorders, rehabilitation, non-invasive brain stimulation, repetitive transcranial magnetic stimulation, transcranial direct current stimulation, virtual reality, brain–computer interface

## Abstract

Cerebral stroke is one of the leading disability causes among adult population worldwide. The number of post-stroke patients, who need rehabilitation including motor recovery, keeps growing annually. Standard motor rehabilitation techniques have a limited effect on recovering extremity motor defunctionalization. In this regard, in recent years, new technologies of post-stroke rehabilitation are being suggested. The present review summarizes the existing literature data on current techniques applied in patients with motor disorders at an early rehabilitation period of cerebral stroke. The current modern technologies are divided into the methods based on “interhemispheric inhibition” theory (repetitive transcranial magnetic stimulation, transcranial direct current stimulation), and on “mirror neurons” theory (virtual reality systems and brain–computer interfaces). The authors present the neurophysiological causes and feasible protocols of using the techniques in clinical practice, the clinical research findings due to the initial severity level of motor disorders and stroke age, as well as the factors contributing to the motor rehabilitation efficiency when using these methods.

## Introduction

Over the period of 1990–2019, the number of annually recorded stroke cases has increased by 70%, amounting to 12.2 million patients per year [1]. Currently, stroke is the third, in order of importance, disability cause in adult population worldwide [1, 2]. The increase in the number of patients requiring neurorehabilitation calls for a medical community to develop and introduce into clinical practice novel rehabilitation techniques to recover impaired motor functions.

One of the paramount tasks of post-stroke rehabilitation is extremity motor function restoration. At least in 50% of patients after stroke, the disability results from motor deficit of upper and/or lower extremities [3, 4]. Post-stroke upper extremity motor disorders are considered more clinically significant and more difficult to recover compared to lower extremity motor deficit [5-8]. That is exactly why the most of the existing clinical studies are likely to focus on motor rehabilitation of patients with upper extremity motor disorders.

For effective neurorehabilitation, the most important is the understanding of interneuronal interactions, as well as neurophysiological aspects of brain tissue damage and repair. The rehabilitation activity of impaired functions due to stroke is known to alter over time [4, 9]. In literature, there are the reports on natural neuroplasticity being particularly marked during the first 3–6 months of stroke [10, 11]. Therefore, the study of using high-tech techniques at an early rehabilitation period (subacute phase of stroke) seems to be a crucial task in modern neurorehabilitation [7, 10].

The usage principles of modern technologies aimed at motor recovery are based on two main neurophysiological theories: 1) interhemispheric inhibition (IHI) theory and 2) mirror neurons (MNs) theory. In particular, repetitive transcranial magnetic stimulation and transcranial direct current stimulation are conventionally referred to motor rehabilitation methods based on interhemispheric inhibition theory. Training techniques in virtual reality and brain–computer interfaces are used to activate the so-called mirror neuron network.

**The aim of the present review** was to summarize and study the relevant information on modern technologies currently applied in patients with motor disorders at an early rehabilitation period of cerebral stroke.

Literature search was conducted in Scopus and Web of Science databases, in the PubMed search system in MEDLINE and PubMed Central, on the Springer Link publisher platform, in BioMed Central, Free Medical Journals, SSRN, and Google Scholar by key words: stroke rehabilitation, motor disorders, non-invasive brain stimulation, repetitive transcranial magnetic stimulation, transcranial direct current stimulation, virtual reality, brain–computer interface.

High-tech motor rehabilitation methods, which can be used in an early post-stroke rehabilitation period, are summarized and represented in the Figure. Further in the text, they are given in detail.

**Figure 1.**
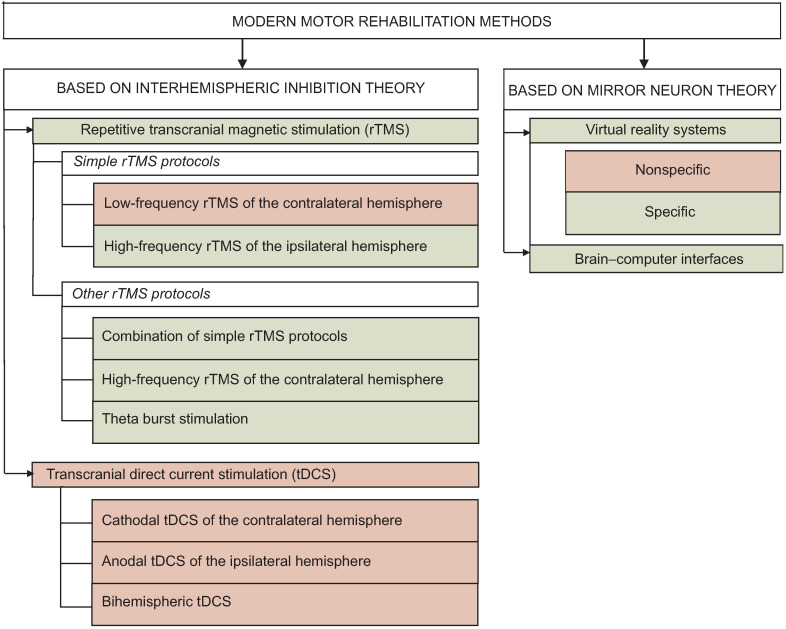
Modern rehabilitation technologies of patients with motor disorders and the efficiency of their usage in an early rehabilitation period of cerebral stroke: 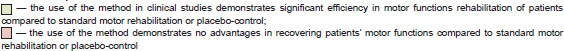

## High-tech methods based on interhemispheric inhibition theory

In the norm, firing neurons of one brain hemisphere are believed to have an inhibitory effect on the neurons of the contralateral hemisphere providing interhemispheric balance and balanced neurophysiological processes. Meanwhile, according to the studies using functional magnetic resonance imaging (fMRI) and diagnostic transcranial magnetic stimulation (TMS), the decrease in the number of functioning neurons in the stroke-affected hemisphere can lead to disequilibrium towards the intact hemisphere and the formation of an excessive inhibitory levels of the affected brain cortex. Moreover, the intensity of interhemispheric asymmetry hyperactivation of primary motor cortex of the intact hemisphere directly correlates with motor deficit severity [12-14] and is considered to be a factor preventing from natural recovery of impaired motor functions [15].

In order to correct interhemispheric asymmetry and improve the rehabilitation efficiency of post-stroke patients with motor deficit, the availability of using in clinical practice some non-invasive methods for brain stimulation is being discussed. Particularly, as adjuvant means of neurorehabilitation, the following techniques are suggested: repetitive transcranial magnetic stimulation (rTMS) and transcranial direct current stimulation (tDCS) [16].

It should be mentioned that currently there is no consensus on the presence of pathological hyperexcitability of the contralateral motor cortex and its negative effect on functional outcomes of patients with post-stroke disorders. Thus, most clinical researches sustain the theory of interhemispheric inhibition [17-19]. However, Xu et al. [20] when using diagnostic TMS recorded the absence of pathological interhemispheric inhibition in an acute and early rehabilitation periods of cerebral stroke, and its occurrence with motor functions improving at a late rehabilitation period. Therefore, the issue on the necessity of using rTMS and tDCS in an early rehabilitation period in order to correct interhemispheric asymmetry remains open. Moreover, the usage efficiency of these neuromodulation techniques in motor post-stroke rehabilitation is not thoroughly studied, which requires further researches.

### Repetitive transcranial magnetic stimulation

Transcranial magnetic stimulation is a method applied to deliver electrical pulses via skin to brain tissues using a magnetic field. TMS can be used both diagnostically and therapeutically [21]. Diagnostic TMS mode is single electrical pulsing; it enables to assess the integrity and functioning of motor path of the nervous system. rTMS is used for therapy and motor neurorehabilitation.

Repetitive transcranial stimulation consists in a continuous series of electrical pulses in brain tissue using a variable magnetic field [15, 22]. Currently, two main rTMS modes are used: high-frequency and low-frequency. The excitability of cerebral neurons is recognised to increase in high-frequency stimulation (3–10 Hz), while in low-frequency (1 Hz) the effect is the opposite [23].

So far, there have been developed several rTMS protocols, which have different neuromodulating effects. Low-frequency rTMS (LF-rTMS) of the contralateral hemisphere and high-frequency rTMS (HF-rTMS) of the ipsilateral hemisphere are referred to simple protocols. Other rTMS protocols are being suggested now as well. They are expected to be more effective to correct motor disorders at an early post-stroke recovery period.

#### Simple rTMS protocols

*Low-frequency rTMS of the contralateral hemisphere* is one of the main and most common rTMS protocols used in motor post-stroke rehabilitation [23]. The method consists in the effect the magnetic field has on the intact cerebral hemisphere to weaken its excessive inhibitory influence. LF-rTMS efficiency and the instance of activation decrease of the contralateral hemisphere when exposed to LF-rTMS were proved in a number of clinical studies using different functional diagnostic techniques: electroencephalography (EEG) [24], fMRI [14], and diagnostic TMS [25].

Most studies [19, 26, 27] report the data on the presence or absence of only mild superiority of the stimulation protocol compared to a placebo group concerning motor function improvement of impaired upper and lower extremities in an early rehabilitation period after stroke (Table 1).

**Table 1:** Efficiency of low-frequency and high-frequency repetitive transcranial magnetic stimulation depending on initial severity of motor disorders and cerebral stroke age

Reference	Protocols	Stroke type and the number of patients	Post-stroke time: mean value/range	Initial severity of motor disorders	Results	Level of evidence
***Low-frequency rTMS of the contralateral hemisphere (upper extremity)***						
Kim et al., 2020 [26]	Group 1 — LF-rTMS, 1 Hz — 100%, 1800 impulses, 10 sessions (5 per week); group 2 — placebo	IS (n=77)	2 weeks/up to 3 months	Moderate upper extremity function abnormality (on average, 40.7 points according to FMA-UE scale)	Clinically significant improvement of upper extremity motor functions according to BBT, FMA-UE scales were found in both groups. No significant differences were revealed between the groups immediately after the therapy and a month later (p=0.267)	1b (A)
Luk et al., 2022 [19]	Group 1 — LF-rTMS, 1 Hz — 90%, 1200 impulses, 10 sessions (5 per week); group 2 — placebo	IS (n=23) HS (n=1)	—/1–6 months	Mild and moderate function abnormality of the upper extremity (on average, 47.8 points according to FMA-UE scale)	Clinically significant improvement of upper extremity motor functions according to FMA-UE, ARAT, and BBT scales were found in both groups. In group 1, there were more marked improvement compared to group 2 according to FMA-UE (p=0.004), ARAT (p=0.002), and BBT (p=0.005) scale findings	1b (A)
*Low-frequency rTMS of the contralateral hemisphere (lower extremity)*						
Huang et al., 2018 [27]	Group 1 — LF-rTMS, 1 Hz — 120%, 900 impulses, 15 sessions (5 per week); group 2 — placebo	IS (n=25) HS (n=13)	1 month/ 10–90 days	Moderate disorders of the lower extremity functions (on average, 12.9 points according to FMA-LE scale)	Clinically significant improvement of lower extremity motor functions according to TUG, FMA-LE, and BI scales were found in both groups. There were found no clinically significant differences between the groups according to TUG, FMA-LE, BI scales data (p>0.05)	1b (A)
*High-frequency rTMS of the ipsilateral hemisphere*						
Guan et al., 2017 [29]	Group 1 — HF-rTMS, 5 Hz — 120%, 1000 impulses, 10 sessions (daily); group 2 — placebo	IS (n=42)	1 week/1–14 days	Moderate function impairments of upper and lower extremities (on average, the score was 39.2 according to FMA-UE scale and 24.9 according to FMA-LE scale)	Clinically significant improvement of upper and lower extremity motor functions according to NIHSS, BI, FMA-UE, FMA-LE scales data were found in both groups. Group 1 was found to have more pronounced improvement according to NIHSS (p=0.032), BI (p=0.047), FMA-UE (p=0.037) scales compared to group 2 immediately after therapy and a month after therapy. According to FMA-LE scale data, there were no differences found between the groups (p=0.952). 3, 6, and 12 months after therapy, the differences between the groups were found only according to FMA-UE scale	1b (A)
Haghighi et al., 2021 [7]	Group 1 — HF-rTMS, 20 Hz — 90%, 2000 impulses, 10 sessions (3 per week); group 2 — placebo	IS/HS (n=20)	3 months/up to 6 months	Moderate function impairments of the upper extremity (the score: 22– 44 points according to FMA-UE scale)	Clinically significant improvement of upper extremity motor functions according to FMA-UE, BBT scales, pinch strength was observed in both groups. In group 1, there was more expressed improvement compared to group 2 according to BBT scale (p=0.003) and grip strength (p=0.007). The comparison of the groups showed the tendency for improving motor functions in group 1 according to FMA-UE (p=0.063) and pinch strength (p=0.353)	1b (A)

Here: LF/HF-rTMS — low-/high-frequency repetitive transcranial magnetic stimulation; IS — ischemic stroke; HS — hemorrhagic stroke; FMA-UE — The Fugl-Meyer Assessment Upper Extremity; FMA-LE — The Fugl-Meyer Assessment Lower Extremity; BBT — Box and Block Test; ARAT — Action Research Arm Test; TUG — Timed Up and Go Test; BI — Barthel Index for activities of daily living; NIHSS — National Institutes of Health Stroke Scale.

When using *high-frequency rTMS of the ipsilateral hemisphere*, the electrodes are applied above М1 area of the ipsilateral cerebral hemisphere. HF-rTMS is considered to act to raise excitability of the remaining neurons of the stroke-affected hemisphere, and it results in the motor cortex reorganization and accelerated recovery rate of lost motor functions. The efficiency of HF-rTMS of the ipsilateral hemisphere has been proved by the studies using fMRI [14] and diagnostic TMS [14, 28]. Generally, there were demonstrated the best functional outcomes for both upper and lower extremities compared to a placebo group [7, 29, 30] (see Table 1).

In addition, according to Du et al. [14], the comparison of the protocols of LF-rTMS of the contralateral hemisphere and HF-rTMS of the ipsilteral hemisphere demonstrated the efficiency of both modes in recovering motor function of an upper extremity. However, more marked motor improvements were found when using high-frequency stimulation of the affected hemisphere.

#### Other rTMS protocols

*A combined protocol of combined usage of LF- and HF-rTMS* can be referred to the relatively new rTMS modes. Particularly, Long et al. [31] compared the efficiency of this protocol (group 1) with the use of LF-rTMS of the contralateral hemisphere (group 2) and placebo simulation (group 3) at an early rehabilitation period after stroke. The authors showed that clinically significant improvement of motor functions was observed in all three groups immediately after therapy and after 3 months after the therapy. The best results were found in group 1.

##### High-frequency rTMS of the contralateral hemisphere

In most cases in post-stroke patients who has an extensive damage of one hemisphere, the use of the above-described rTMS protocols in motor post-stroke rehabilitation failed to be effective. The researchers explain the fact as follows: in a gross unilateral cerebral lesion, interhemispheric inhibition becomes weak, and the number of neurons in ipsilateral hemisphere is not enough to compensate the lost motor functions [32, 33]. Using cerebral fMRI, it has been shown that in the intact (which is contralateral to the lesion) hemisphere, compensatory neuronal connections form promoting the functional recovery of the lost motor functions [32, 34]. In this regard, the patients with a gross unilateral cerebral cortex and marked motor impairments were suggested to undergo HF-rTMS (М1 area of the intact hemisphere) in order to additionally activate the contralateral hemisphere reorganization. rTMS protocol efficiency has drawn its confirmation in clinical researches. Thus, Wang et al. [35] registered clinically significant improvement in patients, who underwent HF-rTMS of the contralateral hemisphere, while the patients with LF-rTMS or placebo simulation showed no positive functional outcomes after stroke.

*Theta burst stimulation (TBS)* refers to the latest neuromodulation protocols based on repetitive TMS. The method consists in the following: a series of electrical stimuli “packets” are delivered to cerebral tissues. The “packets” are repeated every 200 ms (5 Hz frequency) and consist of three impulses given at 20 ms intervals (50 Hz frequency) [36]. It is expected that this stimulation technique can cause the more long-lasting neuroplastic effects compared to other non-invasive brain stimulation techniques [37].

There are three TMS modes suggested: continuous (cTBS), intermittent (iTBS), and intermediate (imTBS) [36, 38]. Continuous TBS is considered to cause the decrease in cortical excitability due to synaptic transmission inhibition. Intermittent TBS, on the contrary, facilitates neurotransmission and induces excitatory effects [36]. Both modes (continuous and intermittent) find their application in clinical researches [39-43]. When using intermediate TBS, there can be reached the balance between inhibition effects and synaptic transmission facilitation, and there is observed no effect on cortex excitability [36]. Therefore, currently, the mode is not used in motor post-stroke rehabilitation.

Generally, there is insufficient evidence of TBS efficiency in motor post-stroke rehabilitation. Not numerous studies carried out so far have demonstrated a positive effect of TBS on neuroplasticity in an acute and early rehabilitation period of cerebral stroke [39, 40, 42, 43].

### Transcranial direct current stimulation

Transcranial electrical stimulation is another common technique of non-invasive brain stimulation used in order to correct interhemispheric asymmetry and improve the rehabilitation efficiency of post-stroke patients with motor disorders. The data has been given on the availability of using the method in three main modes: transcranial direct current stimulation (tDCS), transcranial alternating current stimulation (tACS), and transcranial random noise stimulation (tRNS) [44, 45]. tDCS has obtained the widest circulation in post-stroke rehabilitation. Two other modes (tACS and tRNS) are underinvestigated so far, and they are not used in clinical practice.

In tDCS, there is weak continuous current supply (1.0– 2.5 mA) to cerebral tissues using two electrodes [46]. Currently, there have been used two main tDCS modes: anodal and cathodal. Anodal stimulation causes the depolarization of neuronal membranes and, therefore, the increase in cortical excitability, while under cathodal tDCS the hyperpolarization is created, and the effect is the opposite [44, 46]. The fact that transmembrane difference of potentials shift under anodal and cathodal tDCS was proved by the research by Nitsche et al. [47] using sodium and calcium channel blocker.

The last publications have reported the data on using three possible tDCS protocols in motor post-stroke rehabilitation: anodal stimulation of М1 ipsilateral hemisphere [48, 49], cathodal stimulation of М1 contralateral hemisphere [50], and bihemispheric stimulation of М1 combining the above-mentioned protocols [51]. Neurophysiological bases of the presumed mechanisms of rehabilitation effect of each of the mentioned tDCS protocols are similar to those we considered before for rTMS.

It should be noted that currently there are a few works devoted to the efficiency of tDCS used to contribute to motor neurorehabilitation of post-stroke patients at an early rehabilitation period; the studies do not demonstrate the advantages of the technique over placebo simulation [50-56] (Table 2). However, recent meta-analyses and a systematic review have reported that the best motor outcomes under tDCS were found in patients, whose rehabilitation had started 6 months after stroke development [57-60]. Therefore, it is most appropriate to use tDCS in a late rehabilitation period (chronic stroke patients).

**Table 2:** Efficiency of transcranial direct current stimulation depending on initial severity of motor disorders and cerebral stroke age

Reference	Protocols	Stroke type and the number of patients	Post-stroke time	Initial severity of motor disorders	Results	Level of evidence
Chang et al., 2015 [48]	Group 1 — anodal tDCS of the ipsilateral hemisphere, 2 mA, 10 min, 10 sessions (5 per week); group 2 — placebo	IS (n=24)	7–30 days	Mild impairments of lower extremity function (the ability to walk without support)	Group 1 demonstrated more marked improvement compared to group 2 according to FMA-LE and MI-LE scales. According to FAC and BBS scales, there were no differences between the groups	1b (A)
Klomjai et al., 2018 [51]	Group 1 — bihemi-spheric tDCS (anodal ipsilateral and cathodal contralateral hemisphere), 2 mA, 20 min, 2 sessions (no more than 1 per week); group 2 — placebo	IS (n=19)	Up to 6 months	Mild impairments of lower extremity function (the ability to walk without support at least 3 m)	Group 1 demonstrated more marked improvement compared to group 2 according to FTSTS. According to TUG, there were no differences between the groups	1b (A)
Boasquevisque et al., 2021 [50]	Group 1 — cathodal tDCS of the contralateral hemisphere, 1 mA, 20 min, 6 sessions (3 per week); group 2 — placebo	IS (n=30)	3 days– 6 weeks	Mild, moderate, and severe dysfunctions of the upper extremity (8.0–56.8 points according to FMA-UE scale)	There were found no clinically significant differences between the groups according to FMA-UE, MAL, NIHSS, mRS, and BI scales	1b (A)

Here: tDCS — transcranial direct current stimulation; IS — ischemic stroke; FMA-UE — The Fugl-Meyer Assessment Upper Extremity; FMA-LE — The Fugl-Meyer Assessment Lower Extremity; MI-LE — Lower Extremity Motricity Index; FAC — Functional Ambulatory Category; BBS — Berg Balance Scale; FTSTS — Five-Times-Sit-To-Stand Test; TUG — Timed Up and Go Test; MAL — Motor Activity Log; NIHSS — National Institutes of Health Stroke Scale; mRS — modified Rankin Scale; BI — Barthel Index for activities of daily living.

### Factors influencing motor rehabilitation efficiency when using non-invasive brain stimulation techniques

The literature concerned with motor post-stroke rehabilitation gives contradictory information on a present or absent positive effect when using various non-invasive brain stimulation methods. The ambiguousness of the findings is most likely due to the difference of study designs. On the one hand, the differences can be related to the use of heterogeneous technical characteristics of stimulation and the dependence of a rehabilitation effect on functional state of cerebral neurons; on the other hand — to the criteria required to involve patients in studies, and patients’ individual characteristics.

The technical characteristics are the following: stimulation frequency, intensity, and duration [61], spool orientation, as well as the number of sessions and their ratio [62].

Both technical characteristics and current functional state of neuronal excitability can determine the presence or absence of neuroplasticity induction in non-invasive stimulation. Postsynaptic depolarization level taken in conjunction with exposure time and the dependence of an expected rehabilitation effect on non-invasive stimulation phase is defined as “phase-dependent transcranial stimulation” [63]. Phase-dependent transcranial stimulation presupposes pulse delivery to brain tissues according to a certain phase of ECG sensorimotor rhythm. One of the reflections of functional condition of sensorimotor cortical neurons on ECG is μ-rhythm [64]. μ-oscillations are of asymmetric form, the positive phase area is larger than that of a negative one and associated with the state of low neuronal excitability [64, 65]. When studying electrophysiological peculiarities of higher nervous activity of healthy humans, Baur et al. [65] showed that against the background of accidental stimulation (regardless of the functional state of neuronal excitability and the stimulation phase) М1 and the use of LF-rTMS at the moment of low neuronal excitability (positive μ-rhythm peak on ECG) there occurred inhibiting neurophysiological effects. On the contrary, under LF-rTMS at the moment of high neuronal excitability (negative μ-rhythm peak on ECG) there was observed the tendency to form М1 excitation effects. Thus, the authors conclude that when performing non-invasive cerebral stimulation, to control neuroplasticity induction and effective neurorehabilitation, it is necessary to take into consideration the oscillatory phases of cerebral rhythm and the time period of pulse delivery.

The inclusion criteria for patients, as well as patients’ individual characteristics that are likely to influence the motor post-stroke neurorehabilitation efficiency, include initial severity of dysfunction, the extensiveness and localization of cerebral lesion, non-invasive stimulation promptness, the presence or absence of recorded hemispheric asymmetry [9, 19, 20, 26, 30, 57, 66].

So far, there is no agreement about the necessity and feasibility on using non-invasive brain stimulation in different clinical situations, as well as an optimal choice of the recommended parameters, protocols, and technical characteristics of rTMS and tDCS depending on patients’ individual peculiarities.

## High-tech methods based on “mirror neurons” theory

Currently, the basic neurophysiological theory explaining the mechanisms of virtual reality systems and brain–computer interface is related to the activation of mirror neuron network. The contemporary literature considers three types of mirror neurons: motor, communicative, and emotional [67-69]. It is assumed that the system of “motor” mirror neurons includes a functional group of cells found in different cerebral structures and coordinating the accomplishment of motor and sensor tasks [69]. Among these structures, there are М1, a complementary motor area, dorsal and ventral premotor cortex areas, inferior frontal gyrus, inferior and superior parietal lobules, interparietal sulcus, primary somatosensory cortex, precuneus [67, 70]. Motor mirror neurons demonstrate their electrophysiological activity both when performing or imagining movements and when keeping watching over the task being carried out [28, 69–71]. It is expected that stimulation methods aimed at activating motor mirror neuron network can have a positive effect on neuroplasticity and promote better recovery of motor functions of post-stroke patients’ extremities.

### Virtual reality systems

Virtual reality (VR) systems are based on computer technologies stimulating real environment and providing a user or a patient with a sensation of presence in the reality [72]. A positive effect of VR systems in motor post-stroke neurorehabilitation is considered to be due to the mirror neuron network activation when a patient keeps watching over virtual avatar movements. Moreover, higher activity of using VR systems compared to standard motor rehabilitation can be related to providing and maintaining a high motivation level and patients’ involvement [5, 73].

The fact of neuronal reorganization and neuroplasticity against the background of using VR systems is confirmed by fMRI findings. There has been studied the effect of VR systems on the functional activity of sensorimotor area for both upper and lower extremities. It should be noted that when using VR technologies for upper extremities, there has been recorded the displacement of sensorimotor cortex functional activity from ipsilateral or bilateral to contralateral area [5]. In contrast, the use of VR technologies for lower extremities contributed to bilateral activation of sensorimotor cortex [74]. To a lesser degree, similar functional changes of sensorimotor cortex activity were found when using standard motor rehabilitation in post-stroke patients [27, 75, 76].

It is important to emphasize that most investigations devoted to VR technologies in motor neurorehabilitation were carried out on patients in a late recovery or residual stroke periods [62, 77]. However, the use of VR systems in an early rehabilitation period of stroke demonstrated comparatively better results [62].

VR systems used in motor post-stroke rehabilitation are divided into nonspecific (entertaining videogames) and specific (created exclusively for neurorehabilitation) [78-89] (Table 3).

**Table 3:** Efficiency of nonspecific and specific virtual reality systems depending on initial severity of motor disorders and cerebral stroke age

Reference	Protocols	Stroke type and the number of patients	Post-stroke time: mean value/range	Initial severity of motor disorders	Results	Level of evidence
*Nonspecific VR systems (upper extremity)*						
Saposnik et al., 2016 [81]	Group 1 — VR system based on Wii (Nintendo); group 2 — usual board games (cards, bingo), 60 min, 10 sessions (5 per week)	IS (n=141)	—/up to 3 months	Mild and moderate dysfunctions of the upper extremity (26.1–68.0 s — total time according to WMFT scale)	There were found no clinically significant differences between the groups according to WMFT, BBT, BI scales	1b (A)
Kim et al., 2018 [84]	Group 1 — VR system based on Xbox Kinect (Microsoft); group 2 — placebo, 30 min, 10 sessions (5 per week)	IS (n=16) HS (n=7)	3 weeks/up to 3 months	Mild and moderate dysfunctions of the upper extremity (38 points according to FMA-UE scale)	There were found no clinically significant differences between the groups according to FMA-UE scale (p=0.937)	1b (A)
*Nonspecific VR systems (lower extremity)*						
Cano-Mañas et al., 2020 [80]	Group 1 — VR system based on Xbox Kinect (Microsoft), 20 min, 24 sessions (3 per week); group 2 — standard motor rehabilitation	IS (n=32) HS (n=16)	6 weeks/ 1–6 months	Moderate disorders of lower extremity function (FAC >1): patients are able to maintain steady body position without exterior support	Group 1 demonstrated more marked improvement compared to group 2 according to mRS (p<0.01), BI (p=0.05), POMA (p=0.02), FRT (p<0.01), TUG (p=0.05)	1b (A)
*Specific VR systems (upper extremity)*						
Brunner et al., 2017 [86]	Group 1 — VR system based on YouGrabber (gloves); group 2 — standard motor rehabilitation, 60 min, 20 sessions (5 per week)	IS (n=95) HS (n=25)	3 months/—	Mild, moderate, and severe dysfunctions of the upper extremity	There were found no clinically significant differences between the groups in upper extremity function improvement immediately after therapy (p=0.714) and 3 months after therapy (p=0.777) according to ARAT, BBT, FIM scales	Ib (A)
Wang et al., 2017 [5]	Group 1 — VR system based on Leap Motion; group 2 — standard motor rehabilitation, 45 min, 20 sessions (5 per week)	IS (n=15) HS (n=11)	—/1–6 months	Mild and moderate dysfunctions of the upper extremity	Clinically significant improvement of upper extremity motor function according to WMFT scale (p<0.01) were found in both groups. Group 1 had more marked improvement compared to group 2 (p<0.01)	Ib (A)
Kiper et al., 2018 [89]	Group 1 — VR system with extended biological feedback (RFVE); group 2 — standard motor rehabilitation, 60 min, 20 sessions (5 per week)	IS (n=78) HS (n=58)	3–4 months/ up to 1 year	Moderate disorders of the upper extremity function (the average score was 40.6 according to FMA-UE scale)	Clinically significant improvement of upper extremity motor function according to FMA-UE, FIM, NIHSS, ESAS scales were found in both groups. Group 1 had more marked improvement compared to group 2 according to FMA-UE (p<0.001), FIM (p<0.001), NIHSS (p≤0.014), ESAS (p≤0.022) scales	Ib (A)
de Rooij et al., 2021 [87]	Group 1 — VR system based on Gait (GRAIL); group 2 — standard motor rehabilitation, 30 min, 12 sessions (2 per week)	IS (n=44) HS (n=8)	—/2 weeks– 6 months	Mild and moderate disorders (FAC ≥3)	There were found no clinically significant differences between the groups in the improvement of lower extremity function according to USER-P scale (p=0.22), walking indices, dynamic balance, etc.	Ib (A)

Here: VR system — virtual reality system; IS — ischemic stroke; HS — hemorrhagic stroke; WMFT — Wolf Motor Function Test; FMA-UE — The Fugl-Meyer Assessment Upper Extremity; FAC — Functional Ambulatory Category; BBT — Box and Block Test; BI — Barthel Index for activities of daily living; mRS — modified Rankin Scale; POMA — Tinetti Performance Oriented Mobility Assessment; FRT — Functional Reach Test; TUG — Timed Up and Go Test; ARAT — Action Research Arm Test; FIM — Functional Independence Measure; NIHSS — National Institutes of Health Stroke Scale; ESAS — Edmonton Symptom Assessment System; USER-P — Utrecht Scale for Evaluation of Rehabilitation-Participation.

#### Nonspecific virtual reality systems

Nonspecific VR systems used in motor post-stroke rehabilitation are such commercial gaming systems as Wii (Nintendo, Japan) [79], Xbox Kinect (Microsoft, USA) [80], PlayStation EyeToy (Sony Group Corporation, Japan) [80]. The comparison of their effectiveness with standard motor rehabilitation or usual entertaining games (playing cards, bingo, etc.) showed no significant differences between these rehabilitation techniques in the dynamics of restoring motor functions and motor outcomes [81-84] (see Table 3). The obtained results are consistent with the meta-analysis by Maier et al. [78] published in 2019. The authors concluded the use of nonspecific VR systems to have no significant effect on motor function recovery. Therefore, it is reasonable to use them only outside healthcare facility, at home, in order to increase total rehabilitation time and maintain the motivation.

#### Specific virtual reality systems

Currently, there is a large number of specific VR systems developed intentionally for motor rehabilitation of stroke patients. Among them, there are non-immersive (those not providing complete immersion of a patient in virtual environment) and immersive (VR programmes realized with the help of virtual vision glasses). Specific VR systems use different movement sensors, which in their turn are divided into wearable and non-wearable. Wearable sensors are fixed to the patient’s body (e.g., gloves or exoskeleton), while non-wearable are located in the rehabilitation room [85].

It is necessary to emphasize that currently there is a variety of different specific VR systems. However, it does not seem possible to assess their efficiency in full. There were some large randomized clinical studies with high level of evidence, which recorded no differences between specific VR systems and standard motor rehabilitation [86, 87]. In addition, most researches concerned with the study of clinical efficacy of such systems have demonstrated significant dominance and better functional outcomes compared to standard motor rehabilitation [5, 62, 88–91] (see Table 3).

#### Factors influencing motor rehabilitation efficiency when using virtual reality technologies

VR systems, specific in particular, are considered promising for motor functions recovery in post-stroke patients. Conflicting data on the presence or absence of a positive effect can be due to two groups of causes: firstly, different characteristics of VR system*:* the presence or absence of biological feedback and its intensity [62, 89]; the presence or absence of multisensory stimulation (visual, auditory, tactile) [62]; the presence or absence of increasing over time complexity of assigned motor tasks [80], etc. Secondly, the factors, which can have an effect on VR systems efficiency in motor post-stroke rehabilitation involve the differences in the composition of treatment programs: their promptness, intensity, the number of repetitions, training ratio in virtual reality, and different orientation degree when accomplishing a certain motor task. In particular, Cano-Mañas et al. [80] consider that VR program of motor rehabilitation in an early rehabilitation period of brain stroke should include 3 or more sessions per week, at least within a month, each session lasting not less than 30 min.

Thus, if there are certain characteristics of specific VR systems and literate arrangement of treatment programs using virtual reality technologies, the present modern technique of post-stroke motor rehabilitation is able to have a significant effect on patients’ motor functions recovery compared to standard motor rehabilitation.

### Brain–computer interfaces

Brain–computer interface (BCI) is a system, which enables a user to operate an external device (a robot, exoskeleton, virtual reality) in case of any changes in neuron excitability and imagining the performed movement [92]. BCI use is based on neuropsychological practice with motor patterns. The method presupposes the movement modelling (mental motor trainings) based on prior experience, without any self-contained movements in space. Imagination, as well as the movement itself, activates the network of motor mirror neurons located in brain structures and responsible for motor action formation [70, 71] that is manifested in changing sensorimotor rhythms and can be recorded by ECG [93] or other invasive and non-invasive neurophysiological techniques [94, 95].

At the first stage of using BCI to form a mental motor image, there is the activation of motor areas in brain, and it is received by a BCI detector. At next stage, there occurs the virtual avatar motion, or the simulation of the imaginary motion of the proper extremity is launched by means of exoskeleton or functional electrostimulation [94, 96–100]. Thus, when using BCI a patient has visual contact or proprioceptive feedback resulting in closing the reflex arch of a classical motor action realized due to proper safe efferent and afferent paths [97, 98]. Moreover, it is thought that in using BCI it is possible to recover motor functions by means of activating alternative intact neural networks [94]. Thus, Wu et al. [97] studied brain functional activity in patients in an early rehabilitation period of cerebral stroke before and after neurorehabilitation using BCI. After therapy with fMRI, the authors recorded marked increase in the activity of inter- and intra-hemispheric interactions between different motor cortex areas. Moreover, functional changes were found in sensorimotor, visuospatial, visual areas, and primary auditory cortex, and according to the researchers, it can be related to technical peculiarities of the applied techniques.

It should be noted that currently, there are no large clinical studies with a long-term follow-up period, studying the BCI efficiency in an early stroke rehabilitation period. The participants of most pilot studies were patients in a late stroke rehabilitation period [94, 101]. This was due to the fact that BCI used in earlier periods is considered less safe on account of relative instability and less endurance of post-stroke patients [102]. Meanwhile, few studies devoted to BCI efficiency assessment in patients in an early stroke rehabilitation period demonstrated some advantage of BCI over standard motor rehabilitation [93, 97, 103] (Table 4).

**Table 4:** Efficiency of brain–computer interfaces depending on initial severity of motor disorders and cerebral stroke age

Reference	Protocols	Stroke type and the number of patients	Post-stroke time: mean value/range	Initial severity of motor disorders	Results	Level of evidence
***Brain– computer interface (upper extremity)***						
Wu et al., 2020 [97]	Group 1 — BCI using exoskeleton, 60 min, 20 sessions (5 per week); group 2 — standard motor rehabilitation	IS (n=19) HS (n=6)	2 months/ 1–6 months	Severe disorders of upper extremity functions (on average, 18.43 points according to FMA-UE scale)	Clinically significant improvement of upper extremity motor function according to FMA-UE, ARAT, WMFT scales were found in both groups (p<0.05). Group 1 had more marked improvement by all indices compared to group 2 (p<0.05)	1b (A)
*Brain–computer interface (lower extremity)*						
Zhao et al., 2022 [103]	Group 1 — BCI using virtual reality and a robot set; group 2 — placebo, 30 min, 24 sessions (6 per week)	IS (n=14) HS (n=14)	1 month/2 weeks– 3 months	Severe disorders of lower extremity functions (on average, 10.3 points according to FMA-LE scale; 60.4% patients — FAC=0)	Clinically significant improvement of lower extremity motor function according to FMA-LE, FAC, LOTCA scales was found in both groups. Group 1 had more marked improvement compared to group 2 according to LOTCA scale alone (p=0.049)	1b (A)

Here: BCI — brain–computer interface; IS — ischemic stroke; HS — hemorrhagic stroke; FMA-UE — The Fugl-Meyer Assessment Upper Extremity; FMA-LE — The Fugl-Meyer Assessment Lower Extremity; FAC — Functional Ambulatory Category; ARAT — Action Research Arm Test; WMFT — Wolf Motor Function Test; LOTCA — Loewenstein Occupational Therapy Cognitive Assessment.

In general, BCI use in motor post-stroke rehabilitation has a high potential.

## Conclusion

Modern technologies available for application in motor neurorehabilitation can be divided into the methods based on “interhemispheric inhibition” (repetitive transcranial magnetic stimulation, transcranial direct current stimulation) and those based on “mirror neurons” theory (virtual reality systems and brain–computer interfaces). Currently, high-tech methods used in an early rehabilitation period of cerebral stroke and able to increase the recovery effectiveness of lost extremity motor functions involve most of protocols of repetitive transcranial magnetic stimulation, the use of specific virtual reality systems and brain–computer interfaces. Generally, there are underinvestigated questions of applicability of different modern technologies and the selection of optimal protocols of their usage in the rehabilitation of patients with motor disorders in an early rehabilitation period of cerebral stroke; therefore, additional clinical studies in this field are required.

## References

[1] GBD 2019 Stroke Collaborators (2021). Global, regional, and national burden of stroke and its risk factors, 1990–2019: a systematic analysis for the Global Burden of Disease Study 2019.. Lancet Neurol.

[2] Feigin V.L., Brainin M., Norrving B., Martins S., Sacco R.L., Hacke W., Fisher M., Pandian J., Lindsay P. (2022). World Stroke Organization (WSO): Global Stroke Fact Sheet 2022.. Int J Stroke.

[3] Katan M., Luft A. (2018). Global burden of stroke.. Semin Neurol.

[4] Hatem S.M., Saussez G., Della Faille M., Prist V., Zhang X., Dispa D. (2016). Bleyenheuft Y. Rehabilitation of motor function after stroke: a multiple systematic review focused on techniques to stimulate upper extremity recovery.. Front Hum Neurosci.

[5] Wang Z.R., Wang P., Xing L., Mei L.P., Zhao J., Zhang T. (2017). Leap motion-based virtual reality training for improving motor functional recovery of upper limbs and neural reorganization in subacute stroke patients.. Neural Regen Res.

[6] Twitchell T.E. (1951). The restoration of motor function following hemiplegia in man.. Brain.

[7] Haghighi F.M., Kordi Yoosefinejad A., Razeghi M., Shariat A., Bagheri Z., Rezaei K. (2021). The effect of high-frequency repetitive transcranial magnetic stimulation on functional indices of affected upper limb in patients with subacute stroke.. J Biomed Phys Eng.

[8] Baniqued P.D.E., Stanyer E.C., Awais M., Alazmani A., Jackson A.E., Mon-Williams M.A., Mushtaq F., Holt R.J. (2021). Brain– computer interface robotics for hand rehabilitation after stroke: a systematic review.. J Neuroeng Rehabil.

[9] van Lieshout E.C.C., van der Worp H.B., Visser-Meily J.M.A., Dijkhuizen R.M. (2019). Timing of repetitive transcranial magnetic stimulation onset for upper limb function after stroke: a systematic review and meta-analysis.. Front Neurol.

[10] Dobkin B.H. (2005). Clinical practice. Rehabilitation after stroke.. N Engl J Med.

[11] Grefkes C., Fink G.R. (2020). Recovery from stroke: current concepts and future perspectives.. Neurol Res Pract.

[12] Ward N.S., Cohen L.G. (2004). Mechanisms underlying recovery of motor function after stroke.. Arch Neurol.

[13] Murase N., Duque J., Mazzocchio R., Cohen L.G. (2004). Influence of interhemispheric interactions on motor function in chronic stroke.. Ann Neurol.

[14] Du J., Yang F., Hu J., Hu J., Xu Q., Cong N., Zhang Q., Liu L., Mantini D., Zhang Z., Lu G., Liu X. (2019). Effects of high- and low-frequency repetitive transcranial magnetic stimulation on motor recovery in early stroke patients: evidence from a randomized controlled trial with clinical, neurophysiological and functional imaging assessments.. Neuroimage Clin.

[15] Kobayashi M., Pascual-Leone A. (2003). Transcranial magnetic stimulation in neurology.. Lancet Neurol.

[16] Adeyemo B.O., Simis M., Macea D.D., Fregni F. (2012). Systematic review of parameters of stimulation, clinical trial design characteristics, and motor outcomes in non-invasive brain stimulation in stroke.. Front Psychiatry.

[17] Hummel F.C., Cohen L.G. (2006). Non-invasive brain stimulation: a new strategy to improve neurorehabilitation after stroke?. Lancet Neurol.

[18] Volz L.J., Vollmer M., Michely J., Fink G.R., Rothwell J.C., Grefkes C. (2017). Time-dependent functional role of the contralesional motor cortex after stroke.. Neuroimage Clin.

[19] Luk K.Y., Ouyang H.X., Pang M.Y.C. (2022). Low-frequency rTMS over contralesional M1 increases ipsilesional cortical excitability and motor function with decreased interhemispheric asymmetry in subacute stroke: a randomized controlled study.. Neural Plast.

[20] Xu J., Branscheidt M., Schambra H., Steiner L., Widmer M., Diedrichsen J., Goldsmith J., Lindquist M., Kitago T., Luft A.R., Krakauer J.W., Celnik P.A. (2019). SMARTS Study Group. Rethinking interhemispheric imbalance as a target for stroke neurorehabilitation.. Ann Neurol.

[21] Klomjai W., Katz R., Lackmy-Vallée A. (2015). Basic principles of transcranial magnetic stimulation (TMS) and repetitive TMS (rTMS).. Ann Phys Rehabil Med.

[22] Chervyakov A.V., Poydasheva A.G., Korzhova J.E., Suponeva N.A., Chernikova L.A., Piradov M.A. (2015). Repetitive transcranial magnetic stimulation in neurology and psychiatry.. Zhurnal nevrologii i psihiatrii imeni S.S. Korsakova.

[23] Lefaucheur J.P., Aleman A., Baeken C., Benninger D.H., Brunelin J., Di Lazzaro V., Filipović S.R., Grefkes C., Hasan A., Hummel F.C., Jääskeläinen S.K., Langguth B., Leocani L., Londero A., Nardone R., Nguyen J.P., Nyffeler T., Oliveira-Maia A.J., Oliviero A., Padberg F., Palm U., Paulus W., Poulet E., Quartarone A., Rachid F., Rektorová I., Rossi S., Sahlsten H., Schecklmann M., Szekely D., Ziemann U. (2020). Evidence-based guidelines on the therapeutic use of repetitive transcranial magnetic stimulation (rTMS): an update (2014–2018).. Clin Neurophysiol.

[24] Matsuura A., Onoda K., Oguro H., Yamaguchi S. (2015). Magnetic stimulation and movement-related cortical activity for acute stroke with hemiparesis.. Eur J Neurol.

[25] Du J., Tian L., Liu W., Hu J., Xu G., Ma M., Fan X., Ye R., Jiang Y., Yin Q., Zhu W., Xiong Y., Yang F., Liu X. (2016). Effects of repetitive transcranial magnetic stimulation on motor recovery and motor cortex excitability in patients with stroke: a randomized controlled trial.. Eur J Neurol.

[26] Kim W.S., Kwon B.S., Seo H.G., Park J., Paik N.J. (2020). Low-frequency repetitive transcranial magnetic stimulation over contralesional motor cortex for motor recovery in subacute ischemic stroke: a randomized sham-controlled trial.. Neurorehabil Neural Repair.

[27] Huang Y.Z., Lin L.F., Chang K.H., Hu C.J., Liou T.H., Lin Y.N. (2018). Priming with 1-Hz repetitive transcranial magnetic stimulation over contralesional leg motor cortex does not increase the rate of regaining ambulation within 3 months of stroke: a randomized controlled trial.. Am J Phys Med Rehabil.

[28] Kim J., Yim J. (2018). Effects of high-frequency repetitive transcranial magnetic stimulation combined with task-oriented mirror therapy training on hand rehabilitation of acute stroke patients.. Med Sci Monit.

[29] Guan Y.Z., Li J., Zhang X.W., Wu S., Du H., Cui L.Y., Zhang W.H. (2017). Effectiveness of repetitive transcranial magnetic stimulation (rTMS) after acute stroke: a one-year longitudinal randomized trial.. CNS Neurosci Ther.

[30] Tang Z., Han K., Wang R., Zhang Y., Zhang H. (2022). Excitatory repetitive transcranial magnetic stimulation over the ipsilesional hemisphere for upper limb motor function after stroke: a systematic review and meta-analysis.. Front Neurol.

[31] Long H., Wang H., Zhao C., Duan Q., Feng F., Hui N., Mao L., Liu H., Mou X., Yuan H. (2018). Effects of combining high- and low-frequency repetitive transcranial magnetic stimulation on upper limb hemiparesis in the early phase of stroke.. Restor Neurol Neurosci.

[32] Bajaj S., Housley S.N., Wu D., Dhamala M., James G.A., Butler A.J. (2016). Dominance of the unaffected hemisphere motor network and its role in the behavior of chronic stroke survivors.. Front Hum Neurosci.

[33] McCambridge A.B., Stinear J.W., Byblow W.D. (2018). Revisiting interhemispheric imbalance in chronic stroke: a tDCS study.. Clin Neurophysiol.

[34] Salehi Dehno N., Kamali F., Shariat A., Jaberzadeh S. (2022). Comparison of transcallosal inhibition between hemispheres and its relationship with motor behavior in patients with severe upper extremity impairment after subacute stroke.. J Stroke Cerebrovasc Dis.

[35] Wang Q., Zhang D., Zhao Y.Y., Hai H., Ma Y.W. (2020). Effects of high-frequency repetitive transcranial magnetic stimulation over the contralesional motor cortex on motor recovery in severe hemiplegic stroke: a randomized clinical trial.. Brain Stimul.

[36] Huang Y.Z., Edwards M.J., Rounis E., Bhatia K.P., Rothwell J.C. (2005). Theta burst stimulation of the human motor cortex.. Neuron.

[37] Goldsworthy M.R., Pitcher J.B., Ridding M.C. (2012). The application of spaced theta burst protocols induces long-lasting neuroplastic changes in the human motor cortex.. Eur J Neurosci.

[38] Di Lazzaro V., Pilato F., Dileone M., Profice P., Oliviero A., Mazzone P., Insola A., Ranieri F., Meglio M., Tonali P.A., Rothwell J.C. (2008). The physiological basis of the effects of intermittent theta burst stimulation of the human motor cortex.. J Physiol.

[39] Nicolo P., Magnin C., Pedrazzini E., Plomp G., Mottaz A., Schnider A., Guggisberg A.G. (2018). Comparison of neuroplastic responses to cathodal transcranial direct current stimulation and continuous theta burst stimulation in subacute stroke.. Arch Phys Med Rehabil.

[40] Volz L.J., Rehme A.K., Michely J., Nettekoven C., Eickhoff S.B., Fink G.R., Grefkes C. (2016). Shaping early reorganization of neural networks promotes motor function after stroke.. Cereb Cortex.

[41] Lin L.F., Chang K.H., Huang Y.Z., Lai C.H., Liou T.H., Lin Y.N. (2019). Simultaneous stimulation in bilateral leg motor areas with intermittent theta burst stimulation to improve functional performance after stroke: a feasibility pilot study.. Eur J Phys Rehabil Med.

[42] Meng Y., Zhang D., Hai H., Zhao Y.Y., Ma Y.W. (2020). Efficacy of coupling intermittent theta-burst stimulation and 1 Hz repetitive transcranial magnetic stimulation to enhance upper limb motor recovery in subacute stroke patients: a randomized controlled trial.. Restor Neurol Neurosci.

[43] Nyffeler T., Vanbellingen T., Kaufmann B.C., Pflugshaupt T., Bauer D., Frey J., Chechlacz M., Bohlhalter S., Müri R.M., Nef T., Cazzoli D. (2019). Theta burst stimulation in neglect after stroke: functional outcome and response variability origins.. Brain.

[44] Paulus W. (2011). Transcranial electrical stimulation (tES – tDCS; tRNS, tACS) methods.. Neuropsychol Rehabil.

[45] Klomjai W., Lackmy-Vallée A., Roche N., Pradat-Diehl P., Marchand-Pauvert V., Katz R. (2015). Repetitive transcranial magnetic stimulation and transcranial direct current stimulation in motor rehabilitation after stroke: an update.. Ann Phys Rehabil Med.

[46] Nitsche M.A., Paulus W. (2000). Excitability changes induced in the human motor cortex by weak transcranial direct current stimulation.. J Physiol.

[47] Nitsche M.A., Fricke K., Henschke U., Schlitterlau A., Liebetanz D., Lang N., Henning S., Tergau F., Paulus W. (2003). Pharmacological modulation of cortical excitability shifts induced by transcranial direct current stimulation in humans.. J Physiol.

[48] Chang M.C., Kim D.Y., Park D.H. (2015). Enhancement of cortical excitability and lower limb motor function in patients with stroke by transcranial direct current stimulation.. Brain Stimul.

[49] Bornheim S., Croisier J.L., Maquet P., Kaux J.F. (2020). Transcranial direct current stimulation associated with physical-therapy in acute stroke patients — a randomized, triple blind, sham-controlled study.. Brain Stimul.

[50] Boasquevisque D.D.S., Servinsckins L., de Paiva J.P.Q., Dos Santos D.G., Soares P., Pires D.S., Meltzer J.A., Plow E.B., de Freitas P.F., Speciali D.S., Lopes P., Peres M.F.P., Silva G.S., Lacerda S., Conforto A.B. (2021). Contralesional cathodal transcranial direct current stimulation does not enhance upper limb function in subacute stroke: a pilot randomized clinical trial.. Neural Plast.

[51] Klomjai W., Aneksan B., Pheungphrarattanatrai A., Chantanachai T., Choowong N., Bunleukhet S., Auvichayapat P., Nilanon Y., Hiengkaew V. (2018). Effect of single-session dual-tDCS before physical therapy on lower-limb performance in sub-acute stroke patients: a randomized sham-controlled crossover study.. Ann Phys Rehabil Med.

[52] Lefaucheur J.P., Antal A., Ayache S.S., Benninger D.H., Brunelin J., Cogiamanian F., Cotelli M., De Ridder D., Ferrucci R., Langguth B., Marangolo P., Mylius V., Nitsche M.A., Padberg F., Palm U., Poulet E., Priori A., Rossi S., Schecklmann M., Vanneste S., Ziemann U., Garcia-Larrea L., Paulus W. (2017). Evidence-based guidelines on the therapeutic use of transcranial direct current stimulation (tDCS).. Clin Neurophysiol.

[53] Hesse S., Waldner A., Mehrholz J., Tomelleri C., Pohl M., Werner C. (2011). Combined transcranial direct current stimulation and robot-assisted arm training in subacute stroke patients: an exploratory, randomized multicenter trial.. Neurorehabil Neural Repair.

[54] Achacheluee S.T., Rahnama L., Karimi N., Abdollahi I., Arslan S.A., Jaberzadeh S. (2018). The effect of unihemispheric concurrent dual-site transcranial direct current stimulation of primary motor and dorsolateral prefrontal cortices on motor function in patients with sub-acute stroke.. Front Hum Neurosci.

[55] Chen J.L., Schipani A., Schuch C.P., Lam H., Swardfager W., Thiel A., Edwards J.D. (2021). Does cathodal vs. sham transcranial direct current stimulation over contralesional motor cortex enhance upper limb motor recovery post-stroke? A systematic review and meta-analysis.. Front Neurol.

[56] Elsner B., Kwakkel G., Kugler J., Mehrholz J. (2017). Transcranial direct current stimulation (tDCS) for improving capacity in activities and arm function after stroke: a network meta-analysis of randomised controlled trials.. J Neuroeng Rehabil.

[57] Van Hoornweder S., Vanderzande L., Bloemers E., Verstraelen S., Depestele S., Cuypers K., Dun K.V., Strouwen C., Meesen R. (2021). The effects of transcranial direct current stimulation on upper-limb function post-stroke: a meta-analysis of multiple-session studies.. Clin Neurophysiol.

[58] Chow A.D., Shin J., Wang H., Kellawan J.M., Pereira H.M. (2022). Influence of transcranial direct current stimulation dosage and associated therapy on motor recovery post-stroke: a systematic review and meta-analysis.. Front Aging Neurosci.

[59] Wong P.L., Yang Y.R., Tang S.C., Huang S.F., Wang R.Y. (2022). Comparing different montages of transcranial direct current stimulation on dual-task walking and cortical activity in chronic stroke: double-blinded randomized controlled trial.. BMC Neurol.

[60] Navarro-López V., Del Valle-Gratacós M., Fernández-Matías R., Carratalá-Tejada M., Cuesta-Gómez A., Molina-Rueda F. (2021). The long-term maintenance of upper limb motor improvements following transcranial direct current stimulation combined with rehabilitation in people with stroke: a systematic review of randomized sham-controlled trials.. Sensors (Basel).

[61] Fitzgerald P.B., Brown T.L., Daskalakis Z.J., Chen R., Kulkarni J. (2002). Intensity-dependent effects of 1 Hz rTMS on human corticospinal excitability.. Clin Neurophysiol.

[62] Miclaus R., Roman N., Caloian S., Mitoiu B., Suciu O., Onofrei R.R., Pavel E., Neculau A. (2020). Non-immersive virtual reality for post-stroke upper extremity rehabilitation: a small cohort randomized trial.. Brain Sci.

[63] Zrenner C., Desideri D., Belardinelli P., Ziemann U. (2018). Real-time EEG-defined excitability states determine efficacy of TMS-induced plasticity in human motor cortex.. Brain Stimul.

[64] Pineda J.A. (2005). The functional significance of mu rhythms: translating “seeing” and “hearing” into “doing”.. Brain Res Rev.

[65] Baur D., Galevska D., Hussain S., Cohen L.G., Ziemann U., Zrenner C. (2020). Induction of LTD-like corticospinal plasticity by low-frequency rTMS depends on pre-stimulus phase of sensorimotor μ-rhythm.. Brain Stimul.

[66] Lee J., Lee A., Kim H., Shin M., Yun S.M., Jung Y., Chang W.H., Kim Y.H. (2019). Different brain connectivity between responders and nonresponders to dual-mode noninvasive brain stimulation over bilateral primary motor cortices in stroke patients.. Neural Plast.

[67] Pineda J.A. (2008). Sensorimotor cortex as a critical component of an 'extended' mirror neuron system: does it solve the development, correspondence, and control problems in mirroring?. Behav Brain Funct.

[68] Marshall P.J., Meltzoff A.N. (2011). Neural mirroring systems: exploring the EEG μ rhythm in human infancy.. Dev Cogn Neurosci.

[69] Filimon F., Rieth C.A., Sereno M.I., Cottrell G.W. (2015). Observed, executed, and imagined action representations can be decoded from ventral and dorsal areas.. Cereb Cortex.

[70] Bajaj S., Butler A.J., Drake D., Dhamala M. (2015). Brain effective connectivity during motor-imagery and execution following stroke and rehabilitation.. Neuroimage Clin.

[71] Hardwick R.M., Caspers S., Eickhoff S.B., Swinnen S.P. (2018). Neural correlates of action: comparing meta-analyses of imagery, observation, and execution.. Neurosci Biobehav Rev.

[72] Levin M.F., Weiss P.L., Keshner E.A. (2015). Emergence of virtual reality as a tool for upper limb rehabilitation: incorporation of motor control and motor learning principles.. Phys Ther.

[73] Mekbib D.B., Zhao Z., Wang J., Xu B., Zhang L., Cheng R., Fang S., Shao Y., Yang W., Han J., Jiang H., Zhu J., Ye X., Zhang J., Xu D. (2020). Proactive motor functional recovery following immersive virtual reality-based limb mirroring therapy in patients with subacute stroke.. Neurotherapeutics.

[74] Xiao X., Lin Q., Lo W.L., Mao Y.R., Shi X.C., Cates R.S., Zhou S.F., Huang D.F., Li L. (2017). Cerebral reorganization in subacute stroke survivors after virtual reality-based training: a preliminary study.. Behav Neurol.

[75] James G.A., Lu Z.L., VanMeter J.W., Sathian K., Hu X.P., Butler A.J. (2009). Changes in resting state effective connectivity in the motor network following rehabilitation of upper extremity poststroke paresis.. Top Stroke Rehabil.

[76] Enzinger C., Dawes H., Johansen-Berg H., Wade D., Bogdanovic M., Collett J., Guy C., Kischka U., Ropele S., Fazekas F., Matthews P.M. (2009). Brain activity changes associated with treadmill training after stroke.. Stroke.

[77] Ögün M.N., Kurul R., Yaşar M.F., Turkoglu S.A., Avci Ş., Yildiz N. (2019). Effect of leap motion-based 3D immersive virtual reality usage on upper extremity function in ischemic stroke patients.. Arq Neuropsiquiatr.

[78] Maier M., Rubio Ballester B., Duff A., Duarte Oller E., Verschure P.F.M.J. (2019). Effect of specific over nonspecific VR-based rehabilitation on poststroke motor recovery: a systematic meta-analysis.. Neurorehabil Neural Repair.

[79] Türkbey T.A., Kutlay S., Gök H. (2017). Clinical feasibility of Xbox KinectTM training for stroke rehabilitation: a single-blind randomized controlled pilot study.. J Rehabil Med.

[80] Cano-Mañas M.J., Collado-Vázquez S., Rodríguez Hernández J., Muñoz Villena A.J., Cano-de-la-Cuerda R. (2020). Effects of video-game based therapy on balance, postural control, functionality, and quality of life of patients with subacute stroke: a randomized controlled trial.. J Healthc Eng.

[81] Saposnik G., Cohen L.G., Mamdani M., Pooyania S., Ploughman M., Cheung D., Shaw J., Hall J., Nord P., Dukelow S., Nilanont Y., De Los Rios F., Olmos L., Levin M., Teasell R., Cohen A., Thorpe K., Laupacis A., Bayley M. (2016). Stroke Outcomes Research Canada. Efficacy and safety of non-immersive virtual reality exercising in stroke rehabilitation (EVREST): a randomised, multicentre, single-blind, controlled trial.. Lancet Neurol.

[82] Choi J.H., Han E.Y., Kim B.R., Kim S.M., Im S.H., Lee S.Y., Hyun C.W. (2014). Effectiveness of commercial gaming-based virtual reality movement therapy on functional recovery of upper extremity in subacute stroke patients.. Ann Rehabil Med.

[83] Cannell J., Jovic E., Rathjen A., Lane K., Tyson A.M., Callisaya M.L., Smith S.T., Ahuja K.D., Bird M.L. (2018). The efficacy of interactive, motion capture-based rehabilitation on functional outcomes in an inpatient stroke population: a randomized controlled trial.. Clin Rehabil.

[84] Kim W.S., Cho S., Park S.H., Lee J.Y., Kwon S., Paik N.J. (2018). A low cost Kinect-based virtual rehabilitation system for inpatient rehabilitation of the upper limb in patients with subacute stroke: a randomized, double-blind, sham-controlled pilot trial.. Medicine (Baltimore).

[85] Kim W.S., Cho S., Ku J., Kim Y., Lee K., Hwang H.J., Paik N.J. (2020). Clinical application of virtual reality for upper limb motor rehabilitation in stroke: review of technologies and clinical evidence.. J Clin Med.

[86] Brunner I., Skouen J.S., Hofstad H., Aßmus J., Becker F., Sanders A.M., Pallesen H., Qvist Kristensen L., Michielsen M., Thijs L., Verheyden G. (2017). Virtual reality training for upper extremity in subacute stroke (VIRTUES): a multicenter RCT.. Neurology.

[87] de Rooij I.J.M., van de Port I.G.L., Punt M., Abbink-van Moorsel P.J.M., Kortsmit M., van Eijk R.P.A., Visser-Meily J.M.A., Meijer J.G. (2021). Effect of virtual reality gait training on participation in survivors of subacute stroke: a randomized controlled trial.. Phys Ther.

[88] Xie H., Zhang H., Liang H., Fan H., Zhou J., Lo W.L.A., Li L. (2021). A novel glasses-free virtual reality rehabilitation system on improving upper limb motor function among patients with stroke: a feasibility pilot study.. Med Nov Technol Devices.

[89] Kiper P., Szczudlik A., Agostini M., Opara J., Nowobilski R., Ventura L., Tonin P., Turolla A. (2018). Virtual reality for upper limb rehabilitation in subacute and chronic stroke: a randomized controlled trial.. Arch Phys Med Rehabil.

[90] Rogers J.M., Duckworth J., Middleton S., Steenbergen B., Wilson P.H. (2019). Elements virtual rehabilitation improves motor, cognitive, and functional outcomes in adult stroke: evidence from a randomized controlled pilot study.. J Neuroeng Rehabil.

[91] Rodríguez-Hernández M., Criado-Álvarez J.J., Corregidor-Sánchez A.I., Martín-Conty J.L., Mohedano-Moriano A., Polonio-López B. (2021). Effects of virtual reality-based therapy on quality of life of patients with subacute stroke: a three-month follow-up randomized controlled trial.. Int J Environ Res Public Health.

[92] Graimann B., Allison B., Pfurtscheller G. (2010). Brain– computer interfaces: a gentle introduction. The Frontiers Collection..

[93] Frolov A.A., Mokienko O., Lyukmanov R., Biryukova E., Kotov S., Turbina L., Nadareyshvily G., Bushkova Y. (2017). Post-stroke rehabilitation training with a motor-imagery-based brain-computer interface (BCI)-controlled hand exoskeleton: a randomized controlled multicenter trial.. Front Neurosci.

[94] Su F., Xu W. (2020). Enhancing brain plasticity to promote stroke recovery.. Front Neurol.

[95] Liang W.D., Xu Y., Schmidt J., Zhang L.X., Ruddy K.L. (2020). Upregulating excitability of corticospinal pathways in stroke patients using TMS neurofeedback; a pilot study.. Neuroimage Clin.

[96] Sebastián-Romagosa M., Cho W., Ortner R., Murovec N., Von Oertzen T., Kamada K., Allison B.Z., Guger C. (2020). Brain computer interface treatment for motor rehabilitation of upper extremity of stroke patients — a feasibility study.. Front Neurosci.

[97] Wu Q., Yue Z., Ge Y., Ma D., Yin H., Zhao H., Liu G., Wang J., Dou W., Pan Y. (2020). Brain functional networks study of subacute stroke patients with upper limb dysfunction after comprehensive rehabilitation including BCI training.. Front Neurol.

[98] Biasiucci A., Leeb R., Iturrate I., Perdikis S., Al-Khodairy A., Corbet T., Schnider A., Schmidlin T., Zhang H., Bassolino M., Viceic D., Vuadens P., Guggisberg A.G., Millán J.D.R. (2018). Brain-actuated functional electrical stimulation elicits lasting arm motor recovery after stroke.. Nat Commun.

[99] Bhagat N.A., Yozbatiran N., Sullivan J.L., Paranjape R., Losey C., Hernandez Z., Keser Z., Grossman R., Francisco G.E., O'Malley M.K., Contreras-Vidal J.L. (2020). Neural activity modulations and motor recovery following brain-exoskeleton interface mediated stroke rehabilitation.. Neuroimage Clin.

[100] Kumari R., Janković M.M., Costa A., Savić A.M., Konstantinović L., Djordjević O., Vucković A. (2022). Short term priming effect of brain-actuated muscle stimulation using bimanual movements in stroke.. Clin Neurophysiol.

[101] Ramos-Murguialday A., Curado M.R., Broetz D., Yilmaz Ö., Brasil F.L., Liberati G., Garcia-Cossio E., Cho W., Caria A., Cohen L.G., Birbaumer N. (2019). Brain–machine interface in chronic stroke: randomized trial long-term follow-up.. Neurorehabil Neural Repair.

[102] Hashimoto Y., Kakui T., Ushiba J., Liu M., Kamada K., Ota T. (2022). Portable rehabilitation system with brain-computer interface for inpatients with acute and subacute stroke: a feasibility study.. Assist Technol.

[103] Zhao C.G., Ju F., Sun W., Jiang S., Xi X., Wang H., Sun X.L., Li M., Xie J., Zhang K., Xu G.H., Zhang S.C., Mou X., Yuan H. (2022). Effects of training with a brain-computer interface-controlled robot on rehabilitation outcome in patients with subacute stroke: a randomized controlled trial.. Neurol Ther.

